# Morphologic and morphometric study of the long head of the biceps femoris in male cadavers: tendon and MTJ insights for injury diagnosis

**DOI:** 10.1007/s12565-025-00885-8

**Published:** 2025-07-23

**Authors:** Urszula Emilia Bogacka-Wójcik, Dawid Władysław Dziedzic, Bogdan Mikołaj Ciszek

**Affiliations:** Department of Descriptive and Clinical Anatomy, Medical University of Warsaw, Warsaw, Poland

**Keywords:** Biceps femoris muscle, Proximal tendon, Distal tendon, Proximal attachment, Myotendinous junction

## Abstract

The long head of the biceps femoris (BFlh) is among the most frequently injured components of the hamstring complex, particularly at the myotendinous junctions (MTJs). Despite its clinical relevance, the gross morphology of the BFlh—including its tendon structure and anatomic variability—remains insufficiently characterized. This study aimed to provide a detailed anatomic and morphometric analysis of the BFlh, focusing on the proximal and distal tendons and their integration with surrounding muscle fibers. Thirty-five formalin-fixed male cadavers were dissected via a posterior longitudinal approach. Tendons were subdivided into free, intermediate, and intramuscular portions. Morphometric data were collected using digital calipers and flexible measuring tape, and correlation analyses were performed using appropriate statistical methods. The total muscle length ranged from 42.0 to 50.0 cm. The tendons showed consistent segmentation, with significant correlations between tendon lengths and limb dimensions, as well as between MTJp and MTJd lengths. The thinner, more variable intramuscular segments may contribute to increased injury susceptibility. These findings support a standardized understanding of MTJ architecture and may enhance injury classification, diagnosis, and rehabilitation strategies.

## Introduction

The biceps femoris muscle, a component of the hamstring muscle complex (HMC) is located on the posterior and lateral aspect of the thigh, extending from the ischial tuberosity to the fibular head. It is composed of two anatomically and developmentally distinct heads: the long head (Bflh), associated with the flexor musculature of the lower limb, and the short head (BFsh), related to the gluteal muscle group (Szpinda et al. 2011). The BFlh shares a common proximal origin with the semitendinosus (ST) muscle on the ischial tuberosity (Markee et al. 1955; Terry and LaPrade 1996; Smet and Best 2000; Woodley and Mercer 2005; Rubin 2012; Stępień et al. 2019). The short head (BFsh) originates—directly to the bone—from the lateral lip of the linea aspera and the lateral femoral intermuscular septum (Kolouris and Connell 2005; Carlson 2008; Entwisle et al. 2017).

Hamstring muscles, particularly the long head of the biceps femoris (BFlh), are frequently injured during high-intensity physical activities such as sprinting, jumping, or sudden acceleration and deceleration (Slavotinek et al. 2002; Kolouris and Connell 2005; Mendiguchia et al. 2012; Dolman et al. 2014; Schuermans et al. 2014). Such injuries are prevalent among athletes in sports like soccer, rugby, and track and field (Opar et al. 2012; Ekstrand et al. 2012; Brukner 2015).

The HMC accounts for 10–30% of all sports-related injuries (Orchard and Seward 2002; Petersen and Hölmich 2005; Malliaropoulos 2012; Ekstrand et al. 2012; DeWitt and Vidale 2014; Sandmann et al. 2016), with the BFlh being the most commonly affected—particularly at the myotendinous junction (MTJ) (Crema et al. 2016; Grange et al. 2023).

Despite advances in imaging and clinical studies research, the internal architecture and macroscopic anatomy of the biceps femoris remain poorly characterized in the context of injury mechanisms. A comprehensive understanding of its macroscopic morphology and morphometric parameters—such as tendon lengths, attachment types, and spatial relationships within the muscle belly—is essential for improving diagnostic precision, injury classification, and therapeutic planning. Basic anatomic measurements also support 3D modeling, surgical navigation, and evidence-based rehabilitation strategies.

This study aims to provide a detailed macroscopic and morphometric analysis of the Bflh, focusing on the proximal and distal tendons and MTJs. By examining these structures in cadaveric specimens, the authors aim to clarify anatomic factors influencing injury risk and to support standardization in anatomic and clinical descriptions.

## Materials and methods

### Study population

The study was conducted on 35 long heads of biceps femoris muscles obtained from formalin-fixed male cadavers aged between 23 and 58 years. All specimens were sourced from routine forensic autopsies at the Department of Forensic Medicine, Medical University of Warsaw. The authors hereby confirm that every effort was made to comply with all local and international ethical guidelines and laws concerning the use of human cadaveric donors in anatomic research (ethical review approval number—AKBE/147/2019).

The exclusive use of male specimens was due to their greater availability in the donor pool and to eliminate potential sex-based anatomic variability in muscle–tendon architecture. However, this limitation is acknowledged, and future studies should include both sexes for broader generalizability.

### Sample size and selection criteria

The sample size (*n* = 35) was determined based on the number of intact, usable specimens available over a defined autopsy period. Only specimens with intact posterior thigh regions and no visible signs of musculoskeletal trauma, surgical intervention, or decomposition were included. Cadavers with previous lower limb injuries or deformities were excluded.

### Dissection and morphologic observations

The posterior thigh was dissected using a standard longitudinal approach, extending from the gluteal fold to the popliteal fossa. Skin, subcutaneous tissue, and fascia lata were removed to expose the biceps femoris and adjacent structures. Dissection was performed layer by layer with a scalpel and forceps to preserve muscle–tendon continuity. The long head was isolated to visualize the proximal and distal tendons, including intramuscular segments. Proximal and distal attachments were identified using macroscopic landmarks: the ischial tuberosity and the fibular head, respectively. Due to the unclear margins of the ischial tuberosity, its center was used as the reference point for proximal measurements.

### Morphometric analysis

Measurements were taken using digital calipers with a precision of 1 mm for smaller structures and flexible measuring tapes for overall muscle and tendon lengths. This is critical when working with soft tissues, which are deformable and prone to stretching. Each measurement was taken three times and averaged to minimize inter-observer variability.

**Fig. 1:**
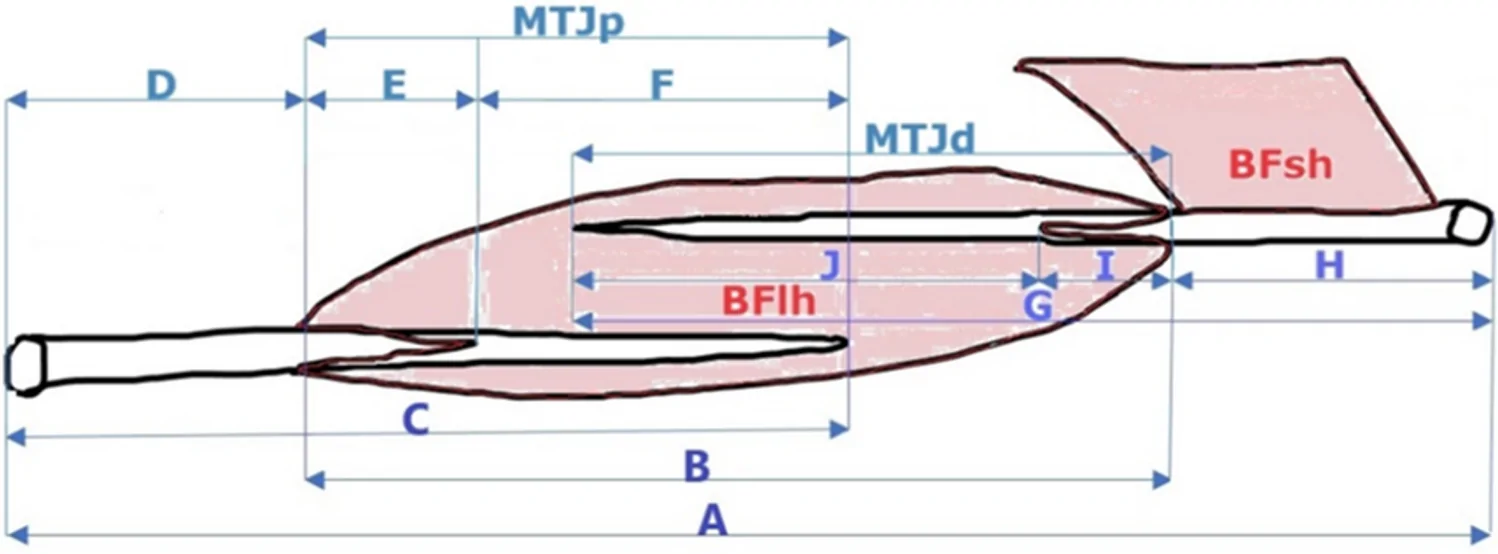
Muscle model with measurement points

The following parameters were recorded (Fig. 1):

- A—total muscle length- measured from the proximal attachment to the distal attachment;
- B—length of the muscular portion of the long head-measured from the proximal portion of muscle fibers in the long head of biceps femoris near its proximal attachment, up to the most distal muscle fibers near the distal attachment;
- C–F—subdivisions of the proximal tendon;
  - C—total length of the proximal tendon: measured from the proximal attachment to the distal end of the tendon within the muscle belly;
  - D—proximal free portion: length of the proximal tendon not covered by muscle fibers;
  - E—proximal intermediate portion: length of the proximal tendon partially covered by muscle fibers, as observed macroscopically;
  - F- proximal intramuscular portion: length of the proximal tendon fully enclosed within the muscle belly;
- G–J—subdivisions of the distal tendon;
  - G—total length of the distal tendon: measured from the distal attachment to the distal end of the tendon within the muscle belly;
  - H—distal free portion: length of the distal tendon not covered by muscle fibers;
  - I—distal intermediate portion: length of the distal tendon partially covered by muscle fibers, as observed macroscopically;
  - J—distal intramuscular portion: length of the distal tendon completely enclosed within the muscle belly;
- MTJp/MTJd—lengths of proximal and distal myotendinous junctions.

### Statistical analysis

Data distribution for each variable was tested using the Shapiro–Wilk test. Based on distribution:

- variables with normal distribution were analyzed using Pearson correlation coefficients.
- variables with non-normal distribution were analyzed using Spearman’s rank correlation.

A *p* value < 0.05 was considered statistically significant. All statistical analyses were performed using Statistica.

## Results

Table 1 presents the results of measurements taken in the lower limbs, as well as in the selected structures of the muscle (Table 1).

**Table 1:** Lengths of selected structures in the biceps femoris muscle

Variable	Descriptive statistics											
	N	M (CM)	Me (cm)	Min (cm)	Max (cm)	Q1 (cm)	Q3 (cm)	Quartile range (cm)	SD (cm)	SE (CM)	SW-W	p
DW	35	89.97	90	80	100	88.5	92	3.5	4.42	0.75	0.965	0.3290
DB	35	82.11	82.5	75	89	80.5	84.5	4	3.33	0.56	0.981	0.7860
DWU	35	52.40	52	45	61	50	54	4	4	0.68	0.954	0.1533
DBU	35	42.19	42	36	49	41	44	3	2.75	0.47	0.968	0.3874
A	35	46.44	46.5	42	50	45	48	3	2.09	0.35	0.94	0.0679
**B**	**35**	**32.54**	**32**	**28.5**	**38.5**	**30**	**35**	**5**	**2.92**	**0.49**	**0.92**	**0.0190**
**C**	**35**	**27.86**	**28**	**24**	**31**	**27**	**29**	**2**	**1.58**	**0.27**	**0.93**	**0.0356**
**D**	**35**	**5.44**	**5.5**	**0**	**9**	**5**	**6.5**	**1.5**	**2.13**	**0.36**	**0.89**	**0.0027**
E	35	15.70	15.5	11	22	14	17	3	2.4	0.41	0.97	0.5450
**F**	**35**	**6.93**	**7.5**	**0**	**12.5**	**5.5**	**9**	**3.5**	**3.12**	**0.53**	**0.91**	**0.0085**
MTJp	35	22.74	22	18	28.5	21	24	3	2.63	0.45	0.95	0.0813
G	35	29.69	29.50	24	35	28	31	3	2.27	0.38	0.977	0.6438
H	35	2.61	2.5	0	6	2	3.5	1.5	1.32	0.22	0.971	0.4629
I	35	24.46	24.5	17.5	31	23	25.5	2.5	2.44	0.41	0.943	0.0684
**J**	**35**	**2.61**	**2.5**	**1**	**9.5**	**1.5**	**2.5**	**1**	**1.81**	**0.31**	**0.618**	**0.0000**
**MTJd**	**35**	**27.13**	**27**	**19**	**35**	**25.5**	**28.5**	**3**	**3.02**	**0.51**	**0.933**	**0.0343**

*DW* relative length of the lower limb, *DB* absolute length of the lower limb, *DWU* relative length of the thigh, *DBU* absolute length of the thigh, *N* number of cases, *M* arithmetic mean, *Me* median, *Min* minimal value, *Max* maximal value, *Q1* lower quartile, *Q3* upper quartile, *SD* standard deviation, *SE* standard error, *SW-W* Shapiro–Wilk statistical test, *p* statistical significance

Results in bold do not meet the criteria of normal distribution (p < 0.05); the remaining abbreviations—see in text

### General muscle measurements

The total length of the long head of the biceps femoris (n = 35) ranged from 42.0 to 50.0 cm, with a mean of 46.4 ± 2.1 cm (Table 1).

### Proximal tendon and proximal myotendinous junction (MTJp)

**Fig. 2:**
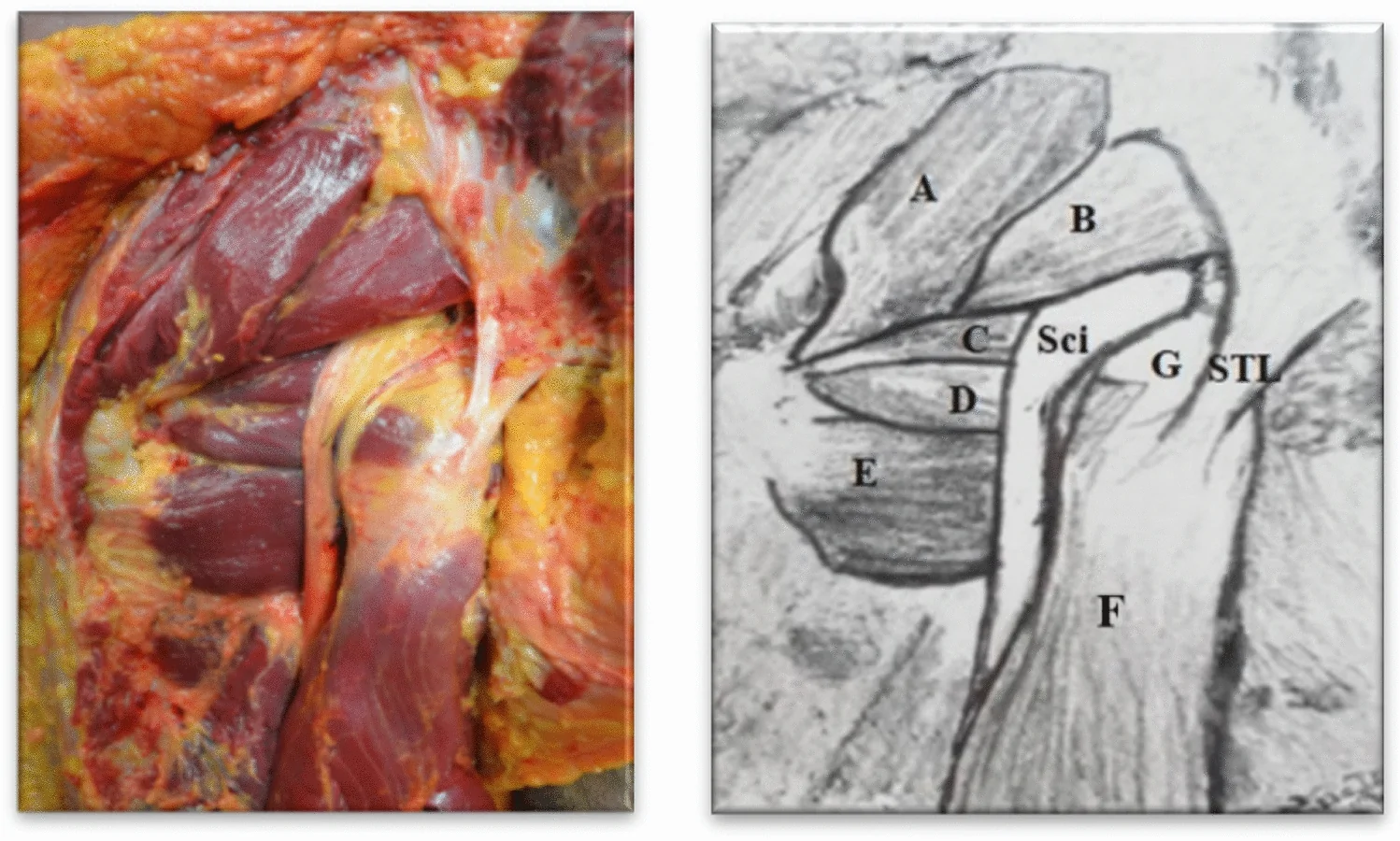
Common proximal attachment of the biceps femoris and the semitendinosus on the ischial tuberosity (G), A—gluteus medius muscle; B—piriformis muscle; C—superior gemellus muscle; D—inferior gemellus muscle; E—quadratus femoris muscle; F—long head of biceps femoris BFlh; STL—sacrotuberous ligament; Sci—sciatic nerve

The proximal tendon originated from the ischial tuberosity, commonly in conjunction with the ST muscle and connected to the part of sacrotuberous ligament (STL) (Fig. 2). Its total length ranged from 24.0 to 31.0 cm, with a mean of 27.9 cm (Table 1).

**Fig. 3:**
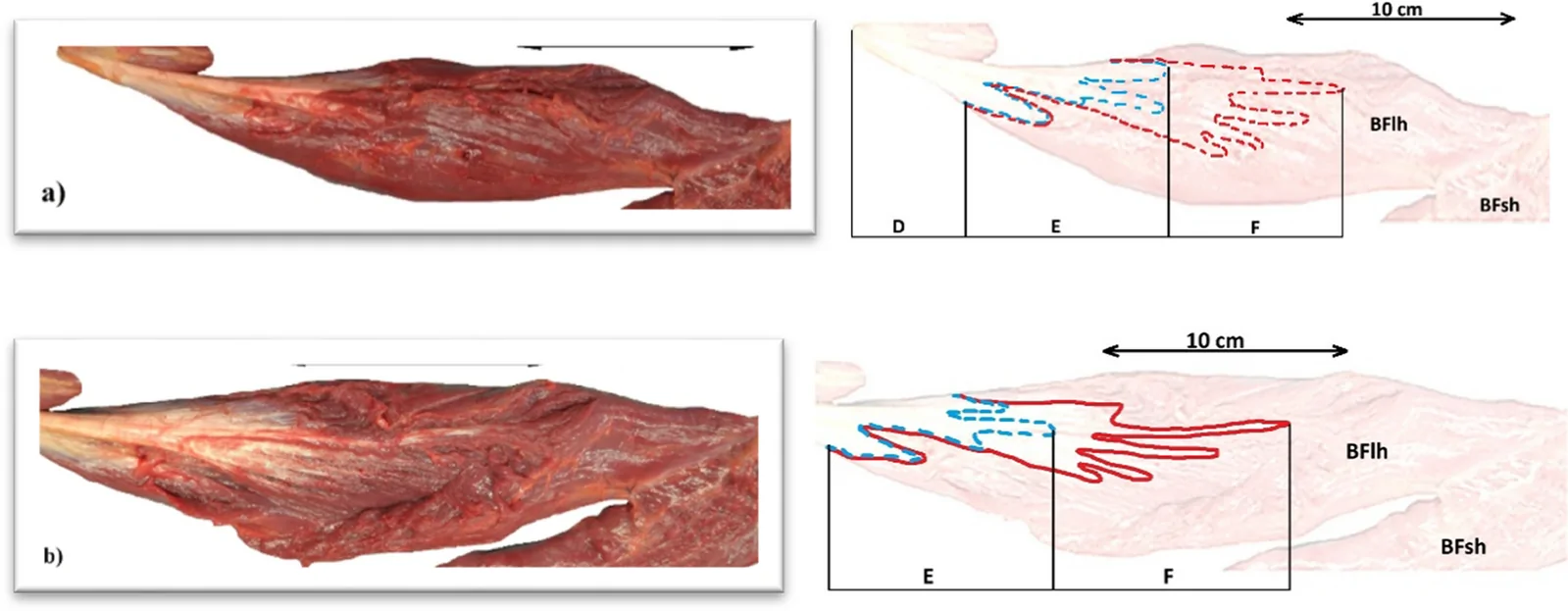
Proximal tendon of the biceps femoris muscle. Divided into two parts based on the proximal tendon’s relationship with long head of biceps femoris muscle fibers. Inferior view of the muscle; BFlh—long head belly; BFsh—short head; D—segment without muscle fibers; E—intermediate part; F—intramuscular part; **a** red dashed line—range of the intramuscular part; **b** blue dashed line—border between the intermediate and intramuscular parts; red line- range of the intramuscular part

The tendon was divided into (Fig. 3a,b) (Table 1):

- proximal free portion (D): not covered by muscle fibers; ranged from 0 to 9.0 cm, mean 5.4 cm;
- proximal intermediate portion (E): partially covered by muscle fibers (flattened plaque-like layer); ranged from 11.0 to 22.0 cm, mean 15.7 cm;
- proximal intramuscular portion (F): fully enclosed within the muscle belly; ranged from 0 to 12.5 cm, mean 6.9 cm (Fig. 3a, b).

**Fig. 4:**
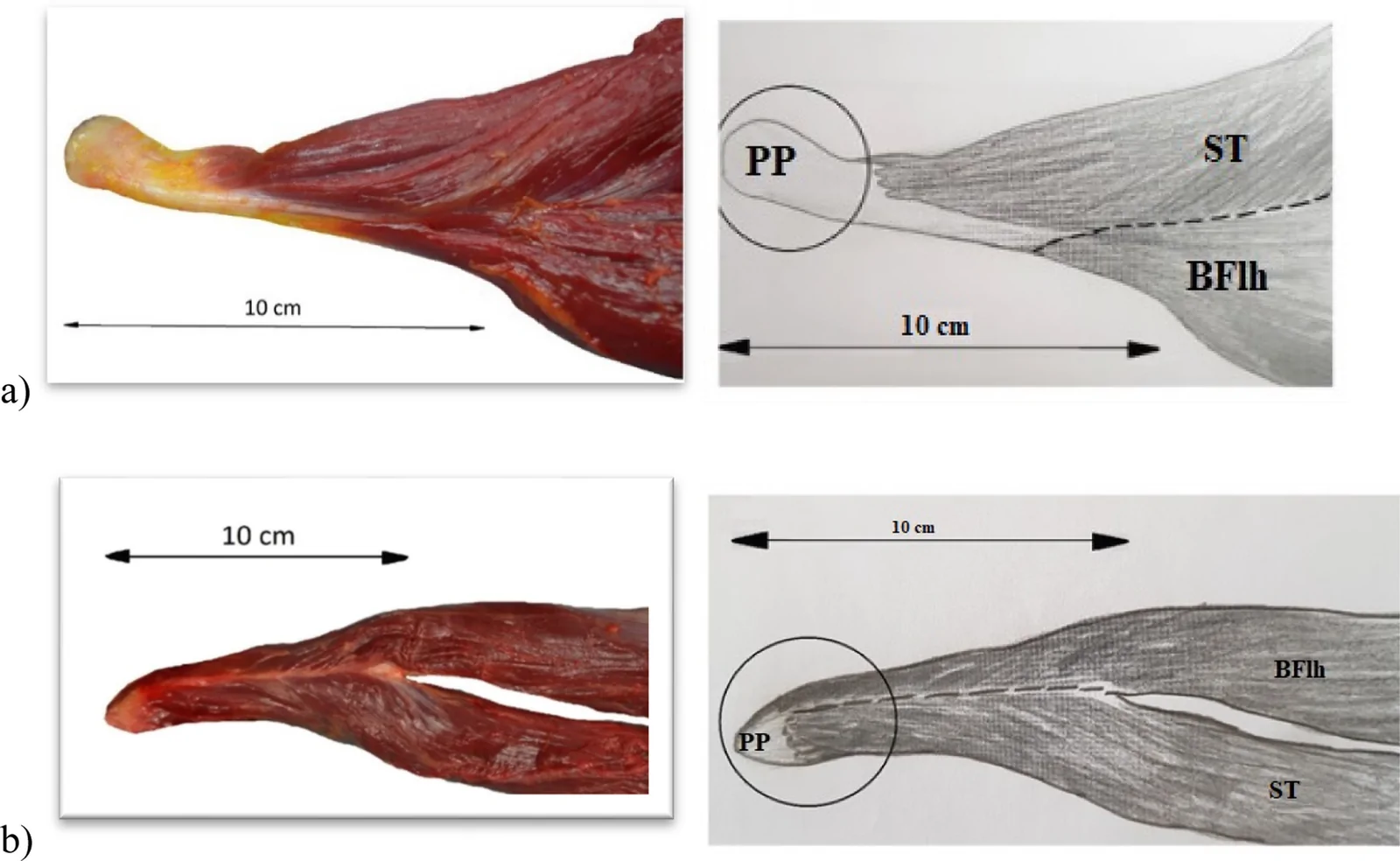
**a, b** Common proximal attachment (PP); ST—semitendinosus muscle fibers; BFlh—muscle fibers of the long head of biceps femoris; dashed line—the border between the long head of biceps femoris muscle fibers and the semitendinosus muscle fibers

In 94.3% of cases the proximal tendon originates with tendinous fibers (Fig. 4a), while in 2 cases (5.7%) muscle fibers were observed within the tendon at its attachment to the ischial tuberosity (Fig. 4b). The total length of the proximal MTJ (MTJp) was calculated by combining E and F.

### Distal tendon and distal myotendinous junction (MTJd)

The distal tendon inserted into the fibular head and was common to both long and short heads of the biceps femoris. Its total length ranged from 17.0 to 32.0 cm, mean 23.5 cm.

**Fig. 5:**
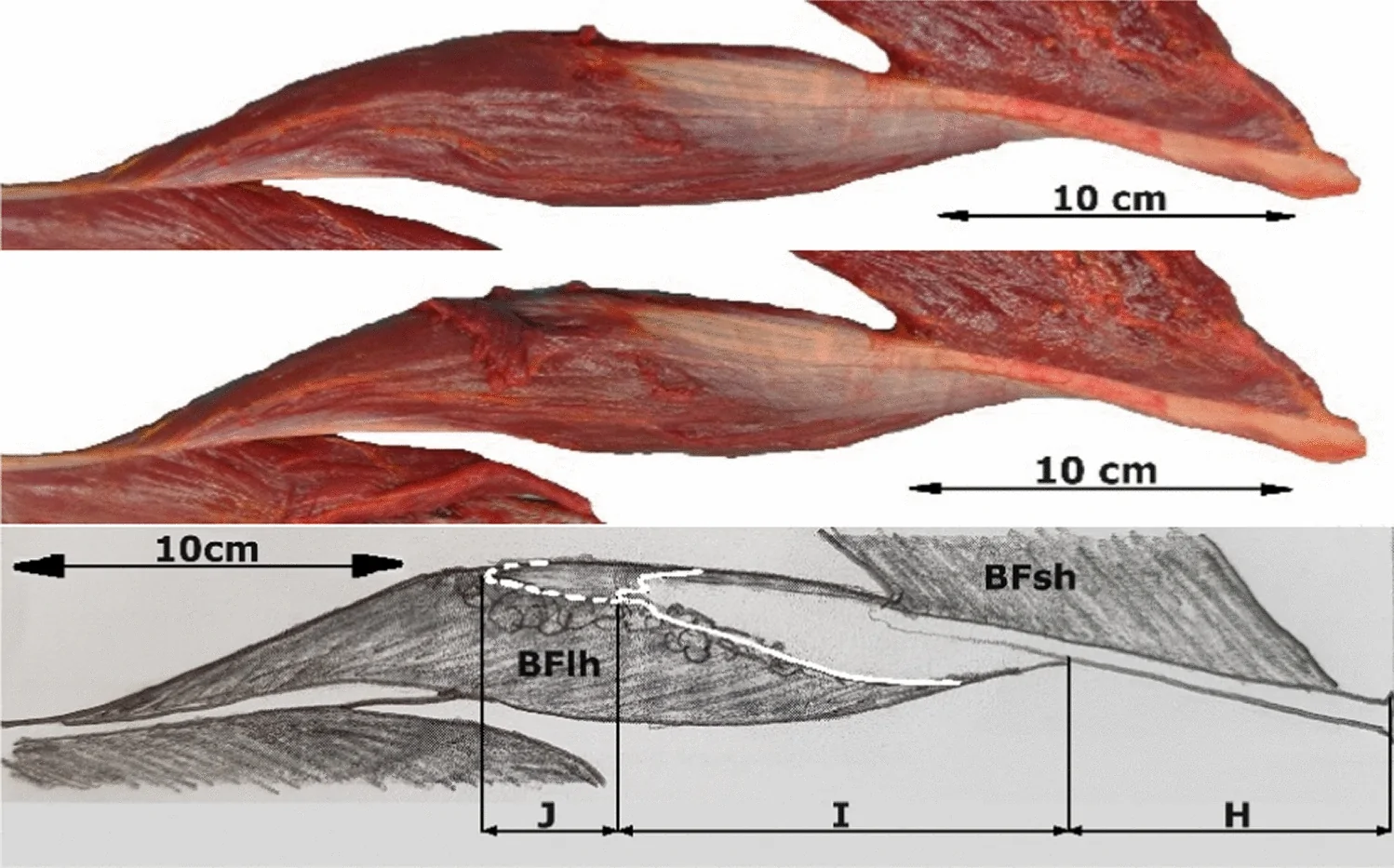
Distal tendon of the biceps femoris muscle. Divided into two parts based on the distal tendon’s relationship with long head of biceps femoris muscle fibers. Inferior view of the muscle; BFlh—long head belly; BFsh—short head; H—segment without muscle fibers; I—intermediate part; J—intramuscular part; white dashed line—range of the intramuscular part; white line—border between the intermediate and intramuscular parts

It was also divided into (Fig. 5) (Table 1):

- distal free portion (H): ranged from 0 to 6.0 cm, mean 2.6 cm;
- distal Intermediate portion (I): partially covered by muscle fibers; ranged from 17.5 to 31.0 cm, mean 24.5 cm;
- distal Intramuscular portion (J): fully embedded within the muscle belly; ranged from 1.0 to 9.5 cm, mean 2.6 cm.

The total length of the distal MTJ (MTJd) included the sum of I and J.

### Correlations between measured parameters

- a strong positive correlation was observed between total muscle length and limb length (Pearson’s *r*, *p* < 0.05);
- the proximal tendon length correlated with both limb length and thigh length (*p* < 0.05);
- the distal tendon showed strong correlations with total muscle length (A), long head length (B), and both MTJp and MTJd lengths;
- a significant correlation was found between the proximal and distal MTJs (*r* = 0.54, *p* < 0.05).
- The proximal MTJ also correlated with:
  - proximal tendon length (C): *r* = 0.49,
  - proximal intramuscular portion (F): *r* = 0.65,
  - and negatively with the proximal free portion (D): *r* = −0.83 (*p* < 0.05);
- the distal MTJ was strongly correlated with total distal tendon length (G): *r* = 0.82 (*p* < 0.05).

All correlation values are presented in Tables 2 and 3. Statistically significant results (*p* < 0.05) are highlighted in italic for clarity.

**Table 2:** Pearson correlation coefficients for variables presenting normal distribution

Variable	The correlation coefficients are significant with *p* < 0.05 N-35									
	A	E	G	H	I	MTJprox	DW	DB	DWU	DBU
A	1.00	−0.02	*0.42*	−0.14	*0.47*	0.15	*0.49*	*0.57*	*0.35*	*0.41*
E	−0.02	1.00	0.26	0.03	0.09	0.18	−0.28	*−0.34*	−0.11	*−0.38*
G	*0.42*	0.26	1.00	*−0.39*	*0.78*	*0.62*	0.12	0.08	0.27	0.05
H	−0.14	0.03	*−0.39*	1.00	*−0.51*	−0.33	0.03	0.27	−0.12	0.29
I	*0.47*	0.09	*0.78*	*−0.51*	1.00	*0.59*	0.19	0.06	0.33	−0.02
MTJprox	0.15	0.18	*0.62*	−0.33	*0.59*	1.00	0.05	−0.06	0.16	−0.01
DW	*0.49*	−0.28	0.12	0.03	0.19	0.05	1.00	*0.85*	*0.91*	*0.80*
DB	*0.57*	*−0.34*	0.08	0.27	0.06	−0.06	*0.85*	1.00	*0.63*	*0.89*
DWU	*0.35*	−0.11	0.27	−0.12	0.33	0.16	*0.91*	*0.63*	1.00	*0.64*
DBU	*0.41*	*−0.38*	0.05	0.30	−0.02	−0.01	*0.80*	*0.89*	*0.64*	1.00

Abbreviations—see in text

**Table 3:** Spearman’s rank correlation

Variable	Spearman rank order correlation; DB removed pairwise, marked correlation coefficients are significant with *p* < 0.05					
	B	C	D	F	J	MTJd
A	*0.41*	0.27	0.01	0.21	−0.15	*0.44*
B	1.00	0.06	*−0.54*	*0.35*	−0.03	*0.56*
C	0.06	1.00	−0.18	*0.49*	−0.08	0.23
D	*−0.54*	−0.18	1.00	*−0.44*	0.02	*−0.37*
E	0.12	0.01	−0.33	*−0.38*	0.33	0.08
F	*0.35*	*0.49*	*−0.44*	1.00	−0.05	*0.47*
G	*0.59*	0.31	*−0.40*	*0.43*	0.33	*0.82*
H	−0.20	0.00	0.12	−0.13	−0.31	*−0.58*
I	*0.5*	*0.38*	*−0.38*	*0.45*	−0.05	*0.83*
J	−0.03	−0.08	0.02	−0.05	1.00	*0.38*
MTJp	*0.51*	*0.49*	*−0.83*	*0.65*	0.11	*0.54*
MTJd	*0.56*	0.23	*−0.37*	*0.47*	*0.38*	1.00
DW	−0.16	*0.45*	0.16	0.22	−0.14	0.09
DB	−0.09	*0.43*	0.28	0.24	−0.28	−0.07
DWU	−0.22	*0.43*	0.07	0.18	0.13	0.23
DBU	−0.10	*0.40*	0.32	0.25	−0.24	−0.15

Abbreviations—see in text

## Discussion

The proximal part of the long head of the biceps femoris (BFlh) is the most commonly affected site of hamstring injuries (Smet and Best 2000; Kolouris and Connell 2005; Battermann et al. 2011; Sato et al. 2012; Malliaropoulos 2012; Mendiguchia et al. 2012; Crema et al. 2016; Azzopardi et al. 2021; Pedret 2021; Grange et al. 2023). Our study confirms classical anatomic descriptions while providing detailed morphologic and morphometric characterization of the BFlh, with particular attention to the proximal attachment, tendon segmentation, and the organization of the MTJs. A detailed understanding of these structures is essential for clinicians dealing with sport-related injuries and reconstructive procedures (James and Moir 1980; Patel et al. 1991; Shanahan et al. 1993, 1997; Rab et al. 1997; Salvador-Sanz et al. 2005; Watanabe et al. 2008; Bertheuil et al. 2013; Onishi et al. 2014).

### Anatomic attachments and tendons structure

Our macroscopic analysis confirmed that the proximal attachment of BFlh is commonly fused with that of the ST, forming a thick, shared tendon that originates on the posteromedial surface of the ischial tuberosity (Battermann et al. 2011; Sato et al. 2012; Storey et al. 2015; Entwisle et al. 2017; Stępień et al. 2019; Azzopardi et al. 2021). The connection between these two muscles was found to be approximately 9.0 cm long (and ranged from 4.5 to 14.5 cm) on the examined specimens, which was also confirmed in the available literature (Battermann et al. 2011) (Table 4). The common part of proximal tendon is defined by most authors as the part of the proximal tendon measured from its attachment to the ischial tuberosity, to the site where the last ST muscle fibers originate.

**Table 4:** Comparison of morphometric data obtained in our research and data from other studies

Structure	Data (cm)		
	Literature data		Own research
	Source	Measurements	Measurements—specimens obtained from adult individuals
Total muscle length (A)	Shanahan et al. 1997)	35–39	46.4 ± 0.4
	Woodley and Mercer (2005)	43.8	
	Van der Made et al. (2015)	42 ± 3.4	
	Tosovic et al. 2016)	46 ± 2.5 measured on ultrasound	
	Kellis et al. 2010)	39.76 ± 3.1	
	Storey et al. 2015)	45.6 ± 2.9 (41.6–50.4)	
Long head muscle belly (B)	Storey et al. 2015)	34.2 ± 2.3 (32–37.9)	32.5 ± 0.5
Proximal tendon (C)	Woodley and Mercer (2005)	27.1	27.9 ± 0.3
	Kellis et al. 2010)	24.03 ± 1.04	
	Van der Made et al. (2015)	19.6 ± 4.1	
	Storey et al. 2015)	25.7 ± 2.9 (20–29.1)	
	Tosovic et al. 2016)	35.5 ± 2.5 measured on ultrasound	
Free part of the proximal tendon (D)	Kellis et al. 2010)	4.93 ± 0.29	5.1 ± 0.4
	Van der Made et al. (2015)	5 ± 3.4	
	Storey et al. 2015)	7.4 ± 1.1 (5.7–9.5)	
Proximal MTJ (MTJp)	Woodley and Mercer (2005)	20.6	22.74 ± 0.45
BFlh and ST common part	Battermann et al. 2011)	9 cm	9 cm (4.5–14.5 cm)
Distal tendon (G)	–	No data for comparison	29.69 ± 0.38
Free part of the distal tendon (H)	–	No data for comparison	2.61 ± 0.22
Distal MTJ (MTJd)	–	No data for comparison	27.13 ± 0.51

Farfán et al. (2021) is the only one author who divided this part into two segments: common attachment measuring 6.7 ± 1.2 cm and the muscle union with ST measuring 3.2 ± 1.4 cm- a proximal tendon segment where ST muscle fibers originate. After adding all these measurements, the results are similar to data presented in other studies, as well as in our research. In the majority of examined specimens, the proximal attachment presented a tendinous structure (94.3%). In 5.7% of cases, muscle fibers were observed within the tendon exactly at its attachment. The shape of the attachment site on the ischial tuberosity was confirmed as oval, aligning with Miller et al. (2007), Freucht et al. (2015), Philippon et al. (2015) and Stępień et al. (2019).

Macroscopic examination also suggests that the proximal attachment of the long head of biceps femoris is connected to the STL, a connection that has been suggested by multiple authors (Markee et al. 1955; Woodley and Mercer 2005; Carlson 2008; Tyler et al. 2010; Storey et al. 2015). Histologic analyses conducted by Sato et al. (2012) confirmed the connection of the STL only with the proximal portion of the attachment belonging to BFlh. The STL demonstrates continuous structure with the common proximal tendon of BFlh and ST, which could explain their limited retraction when their attachment is completely separated from the bone (Bierry et al. 2013).

### Proximal tendon

The proximal tendon was clearly divided into three portions:

- the free portion (D), thick and not covered by muscle fibers;
- the intermediate portion (E), partially covered by muscle fibers and thinner;
- the intramuscular portion (F), delicate and embedded within the muscle belly—resembling a “tendinous core” (Czyrny 2012).

The proximal tendon extended deep into the muscle belly, consistent with the concept of a tendinous core (Czyrny 2012). This central tendon facilitates elastic force transmission and may influence strain patterns (Linklater et al. 2010).

The observed segmentation—particularly the transition from thick, strong free tendons to thin, delicate intramuscular portions—may explain injury susceptibility. These structural transitions require further biomechanical evaluation (Brukner 2015; Tosovic et al. 2016).

### Distal tendon and MTJd

The distal tendon, shared by both heads of the biceps femoris, displayed a similar three-part organization:

- the free portion (H);
- the intermediate portion (I), was relatively long but thinner than the free portion;
- the intramuscular portion (J), which was shorter and thinner than the proximal counterpart but maintained consistent architecture. While few studies report morphometric data for the distal tendon, our findings add original data in this area (Table 4).

### Morphometric correlations and functional implications

Total muscle length showed significant correlation with lower limb length. The proximal tendon correlated with limb and thigh lengths, suggesting adaptation to individual somatotypes (Rubin 2012; Herrmann et al. 2020). Conversely, the distal tendon showed strong associations with internal muscle architecture, possibly reflecting adaptation to mechanical loading (Silder et al. 2008; Linklater et al. 2010).

A noteworthy finding was the significant correlation between MTJp and MTJd lengths, suggesting functional interdependence along the length of the muscle. Physical exertion increases the MTJ interface (Pimentel Neto et al. 2020), which functions as a force transmission zone between muscle and tendon. The correlation between MTJp and MTJd lengths may reflect a functional balance throughout the muscle (Charvet et al. 2012; Tong et al. 2024).

These structural differences—between the thick, free tendon and the thinner intramuscular segment—may affect stress distribution and injury susceptibility. The deep overlap of the proximal and distal tendons, extending over half the muscle length, may support elasticity and efficient force transmission (Linklater et al. 2010).

### Clinical relevance and standardization

Our findings help address limitations in current injury classification systems, particularly regarding the identification of proximal versus distal MTJ injuries. The standardized anatomic terminology and morphometric data presented here can improve diagnostic imaging, guide surgical approaches, and refine rehabilitation protocols. Future research should extend this work with larger, mixed-sex samples and dynamic imaging techniques (Malliaropoulos 2012; Ekstrand et al. 2012; Woods et al. 2004).

## Conclusion

This study confirms classical descriptions of BFlh anatomy while providing novel insight into tendon segmentation, MTJ architecture, and their relationships to body dimensions. These findings contribute to a more consistent anatomic framework for understanding and managing muscle injuries, particularly in athletic populations. The morphometric patterns identified here support the refinement of injury classification systems and offer valuable applications in biomechanical modeling, surgical procedures, and rehabilitation planning.

## Data Availability

The data supporting the findings of this study are available from the corresponding author, Urszula Bogacka-Wójcik, upon reasonable request at urszula.bogacka@wum.edu.pl.

## Availability of data and materials

The datasets used and/or analyzed during the current study are available from the corresponding author on reasonable request.
